# Enhanced Two-Factor Authentication and Key Agreement Using Dynamic Identities in Wireless Sensor Networks

**DOI:** 10.3390/s151229767

**Published:** 2015-11-30

**Authors:** I-Pin Chang, Tian-Fu Lee, Tsung-Hung Lin, Chuan-Ming Liu

**Affiliations:** 1 Department of Digital Applications, Kang Ning University, Tainan 70970, Taiwan; ipin@ukn.edu.tw; 2 Department of Medical Informatics, Tzu Chi University, No. 701, Zhongyang Road, Sec. 3, Hualien 97004, Taiwan; 3 Department of Computer Science and Information Engineering, National Chin-Yi University of Technology, Taichung 41170, Taiwan; duke@ncut.edu.tw; 4 Department of Computer Science and Information Engineering, National Taipei University of Technology, Taipei 10608, Taiwan; cmliu@csie.ntut.edu.tw

**Keywords:** authentication, key agreement, dynamic identity, wireless sensor networks, password, smartcard

## Abstract

Key agreements that use only password authentication are convenient in communication networks, but these key agreement schemes often fail to resist possible attacks, and therefore provide poor security compared with some other authentication schemes. To increase security, many authentication and key agreement schemes use smartcard authentication in addition to passwords. Thus, two-factor authentication and key agreement schemes using smartcards and passwords are widely adopted in many applications. Vaidya *et al.* recently presented a two-factor authentication and key agreement scheme for wireless sensor networks (WSNs). Kim *et al.* observed that the Vaidya *et al.* scheme fails to resist gateway node bypassing and user impersonation attacks, and then proposed an improved scheme for WSNs. This study analyzes the weaknesses of the two-factor authentication and key agreement scheme of Kim *et al.*, which include vulnerability to impersonation attacks, lost smartcard attacks and man-in-the-middle attacks, violation of session key security, and failure to protect user privacy. An efficient and secure authentication and key agreement scheme for WSNs based on the scheme of Kim *et al.* is then proposed. The proposed scheme not only solves the weaknesses of previous approaches, but also increases security requirements while maintaining low computational cost.

## 1. Introduction

### 1.1. Authentication and Key Agreement for WSNs 

**Figure 1:**
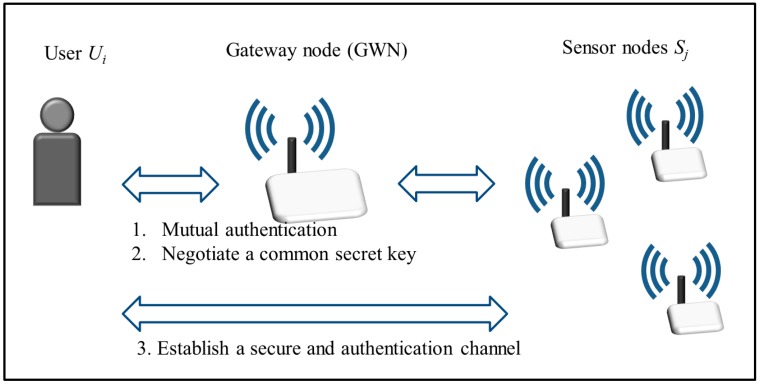
An authentication and key agreement scheme for WSNs.

An authentication and key agreement scheme for WSNs comprises users, sensor nodes and a gateway node (GWN), and enables a user and sensor nodes to realize mutual authentication and to negotiate a common secret key via the help of the GWN. The legitimate user and sensor nodes then establish a secure and authentication channel [1,2,3,4,5,6,7,8,9], as shown in Figure 1. A password-based authentication and key agreement scheme only uses a weak password for user authentication, and is the most convenient authentication method. However, these schemes tend to suffer from some possible attacks, and thus have poor security. To improve security, many authentication and key agreement schemes supplement password authentication with long-term secret keys stored in RFID tags or smartcards [1,8,9,10,11,12]. Since long-term secret keys are not easy to guess and break, two-factor authentication schemes that realize identification using passwords and smartcards may increase security, and thus are suitable for WSNs.

Several efficient two-factor authentication and key agreement schemes for WSNs have been presented recently. For example, in 2009 Das proposed a two-factor authentication and key agreement scheme using passwords and smartcards [1]. The scheme of Das has low computational cost, and is suitable for resource-constrained WSNs. Many improved authentication and key agreement schemes [9,10,11,12,13] were proposed later to solve the security weaknesses in the Das scheme. Yeh *et al.* Chen and Shih [11] in 2010 provided an improved scheme based on the Das scheme to ensure that a legal user can use a WSN in a public environment. Yeh *et al.* [14] in 2011 presented a user authentication scheme based on Elliptic Curves Cryptography (ECC) to overcome the perceived security weaknesses of the scheme of Chen and Shih [11]. However, the scheme of Yeh *et al.* [14] requires time-consuming scalar multiplications on an elliptic curve, and is still insecure against several possible types of attack, and thus fails to provide a secure and efficient solution for WSNs. Vaidya *et al.* [15] in 2012 showed that the Das scheme and its derivatives not only have security flaws, but also do not provide key agreement. Additionally, Kim *et al.* [16] pointed out in 2014 that the scheme of Vaidya *et al.* fails to resist gateway node bypassing and user impersonation attacks, and also proposed an improved scheme that eliminates such security weaknesses and is efficient in term of computational and communication cost. However, their scheme still fails to withstand some possible attacks, as any legitimate user can obtain the secret keys of sensor nodes such that an adversary can perform impersonation, lost smartcard and man-in-the-middle attacks. Moreover, their scheme violates session key security, and fails to provide user privacy protection.

### 1.2. Our Contributions

This investigation presents an efficient and secure authentication and key agreement scheme for WSNs to address the weaknesses of the two-factor scheme of Kim *et al.* [16]. The proposed scheme protects user privacy by using dynamic identities, and by eliminating constant parameters in request messages. Our scheme also encrypts the communicating messages with temporary secret keys rather than constant secret keys of users and sensor nodes, and diminishes redundant variables to ensure session key security. It overcomes the weaknesses in previous schemes, increases security requirements and maintains low computational cost.

### 1.3. Organization of the Paper

The remainder of this investigation is organized as follows: Section 2 lists the notations and definitions adopted in this investigation, reviews the two-factor authentication and key agreement scheme for WSNs of Kim *et al.* [16], and analyzes its weaknesses. Section 3 presents the proposed authentication and key agreement scheme using dynamic identities for WSNs. Section 4 and Section 5 present the results of the security and performance evaluation, respectively. Finally, Section 6 draws the conclusions.

## 2. Preliminaries

This section lists the notations adopted in this paper, describes the underlying primitives used in this investigation, briefly reviews the two-factor authentication and key agreement scheme for WSNs of Kim *et al.* [16], and then addresses the weaknesses of the their scheme.

Assume that *U_i_* denotes the *i*th user; *S_j_* denotes the *j*th sensor node, and *GWN* denotes the gateway node in which *U_i_* and *S_j_* are registered. Table 1 lists the notations used throughout this paper.

**Table 1:** Notation.

*ID_i_*, *pw_i_*	Identity and password pair of user *U_i_*
*SID_j_*	Identity of sensor node *S_j_*
*ID_s_*	Identity of smart card
*K*	Secret key only know to *GWN*
*x_s_*	Secret value of *GWN* and *S_j_*
*K_S_*	Session key
*RN_i_*, *RN_j_*, *RN_G_*	Random numbers selected by *U_i_*, *S_j_* and *GWN*, respectively
*T_i_*, *T_i_′*, *T_j_*, *T_G_*, *T_G_′*	The timestamp values
*h*(·)	A collision free one-way hash function
*f(x, k)*	Pseudo-random function of variable *x* with key *k*
*A* →*B:M*	*A* sends message *M* to *B* through a common channel.
*A* ⇒ *B:M*	*A* sends message *M* to *B* through a secure channel
⊕	The exclusive-or (XOR) operation.
*M*_1_||*M*_2_	Message *M*_1_ concatenates to message *M*_2_.

### 2.1. Review of the Authentication and Key Agreement Scheme of Kim et al.

Kim *et al.* [16] in 2014 proposed an improved two-factor authentication and key agreement scheme for WSNs. Their improved scheme comprises registration, login, authentication and key agreement, and password change phases, which are described as follows:

#### 2.1.1. Registration Phase

In the registration phase, *U_i_* registers his/her identity and password to *GWN*. Then, *GWN* personalizes a smartcard for *U_i_*. Meanwhile, *S_j_* keeps (*SID_j_*, X_S_j__^*^) in its storage before being deployed, where X_S_j__^*^ = *h*(*SID_j_*||*x_s_*):

Step 1: *U_i_* ⇒ *GWN*:{*ID_i_*, *HPW_i_*}
  *U_i_* selects *ID_i_*, password *pw_i_*, a random number *RN_r_*, computes *HPW_i_* = *h*(*pw_i_*||*RN_r_*) and sends {*ID_i_*, *HPW_i_*} to *GWN* via a secure channel.
Step 2: *GWN* ⇒ *U_i_*: *U_i_*′s smartcard
  *GWN* computes *HID_i_* = *h*(*ID_i_*||*K*), *X_S_i__* = *h*(*HID_i_*||*x_s_*), *A_i_* = *h*(*HPW_i_*||*X_S_i__*) ⊕ *h*(*HID_i_*||*K*), *B_i_* = *h*(HPW_i_^*^ ⊕ *X_S_i__*), *C_i_* = *X_S_i__* ⊕ *h*(*ID_s_*||*HPW_i_*) and personalizes the smart card for *U_i_* with the parameters: (*ID_s_*, *HID_i_*, *h*(·), *A_i_*, *B_i_*, *C_i_*). Then, *GWN* sends the smartcard to *U_i_* via a secure channel.
Step 3: *U_i_* computes *XPW_i_* = *h*(*pw_i_*) ⊕ *RN_r_* and inserts *XPW_i_* into his/her smart card.

#### 2.1.2. Login Phase

Step 1: *U_i_* inserts his/her smart card into a terminal and enters ID_i_^*^ and PW_i_^*^.
Step 2: The smart card computes RN_r_^*^ = *h*(*pw_i_*) ⊕ *XPW_i_*, HPW_i_^*^ = *h*(pw_i_^*^||RN_r_^*^), X_S_i__^*^ = *C_i_* ⊕ *h*(*ID_s_*||HPW_i_^*^), B_i_^*^ = *h*(HPW_i_^*^ ⊕ X_S_i__^*^) and verifies B_i_^*^ = ? *B_i_*. If unsuccessful, the smart card aborts this request; otherwise, the smartcard computes *DID_i_* = B_i_^*^ ⊕ *h*(X_S_i__^*^||*RN_i_*||*T_i_*), *M_U_i_,G_* = *h*(*A_i_*||X_S_i__^*^||*RN_i_*||*T_i_*) and *v_i_* = *RN_i_* ⊕ X_S_i__^*^, where *RN_i_* is a nonce and *T_i_* is the current timestamp. Then the smartcard sends the authentication request {*DID_i_*, *M_U_i_,G_*, *v_i_*, *T_i_*, *HID_i_*} to *GWN*.

#### 2.1.3. Authentication and Key Agreement Phase 

This phase enables *U_i_* and *S_j_* to authenticate each other and to negotiate a secret key, and functions as follows:

Step 1: *GWN* → *S_j_*: {*DID_i_*, *M_G,S_j__*, *T_G_*}
  *GWN* checks the validity of *T_i_*, computes *X_S_i__* = *h*(*HID_i_*||*x_s_*), *RN_i_* = *v_i_* ⊕ *X_S_i__*, X^*^ = *DID_i_* ⊕ *h*(*X_S_i__*||*RN_i_*||*T_i_*), M_U_i_,G_^*^ = *h*((X^*^ ⊕ *h*(*HID_i_*||*K*)||*X_S_i__*||*RN_i_*||*T_i_*) and checks M_U_i_,G_^*^ = ? *M_U_i_,G_*. If successful, *GWN* computes *X_S_j__* = *h*(*SID_j_*||*x_s_*), *M_G,S_j__* = *h*(*DID_i_*||*SID_j_*||*X_S_j__*||*T_G_*) and sends {*DID_i_*, *M_G,S_j__*, *T_G_*} to *S_j_*, where *S_j_* is the nearest sensor node for *U_i_* and *T_G_* is current timestamp.
Step 2: *S_j_* → *GWN*: {*y_j_*, *M_S_j_,G_*, *T_j_*}
  *S_j_* checks the validity of *T_G_*, computes M_G,S_j__^*^ = *h*(*DID_i_*||*SID_j_*||X_S_j__^*^||*T_G_*) and checks M_G,S_j__^*^ = ? *M_G,S_j__*. If successful, *S_j_* computes *y_j_* = *RN_j_* ⊕ X_S_j__^*^, *z_i_* = M_G,S_j__^*^ ⊕ *RN_j_* and *M_S_j_,G_* = *h*(*z_i_*||X_S_j__^*^||*T_j_*), *K_S_* = *f*((*DID_i_*||*RN_j_*), X_S_j__^*^), and sends {*y_j_*, *M_S_j_,G_*, *T_j_*} to *GWN*, where *RN_j_* is a nonce and *T_j_* is current timestamp.
Step 3: *GWN* → *U_i_*: {*y_i_*, *w_i_*, *M_G_*_,_*_U__i_*, *q_j_*, *T_G_*′}
  *GWN* checks the validity of *T_j_*, computes *RN_j_* = *y_j_* ⊕ *X_S_j__*, z_i_^*^ = M_G,S_j__^*^ ⊕ *RN_j_*, *M_S_j__*,G^*^ = *h*(z_i_^*^||*X_S_j__*||*T_j_*), and checks M_S_j_,G_^*^ = ? *M_S_j_,G_*. If successful, *GWN* computes *M_G,U_i__* = *h*(*DID_i_*||*M_S_j_,G_*|| *M_U_i_,G_*||*X_S_j__*||*T_G_*′), *w_i_* = z_i_^*^ ⊕ *X_S_i__*, *y_j_* = *RN_j_* ⊕ *X_S_j__*, *q_j_* = *X_S_j__* ⊕ *RN_j_* and sends {*y_i_*, *w_i_*, *M_G_*_,_*_U_i__*, *q_j_*, *T_G_*′} to *U_i_*, where *T_G_*′ is current timestamp.
Step 4: The smart card checks the validity of *T_G_*′ and computes *RN_j_* = *y_j_* ⊕ *X_S_j__*, z_i_^*^ = *w_i_* ⊕ *X_S_i__*, *M_G,S_j__* = z_i_^*^ ⊕ *RN_j_*, M_G,U_i__^*^ = *h*(*DID_i_*||M_S_j_,G_^*^|| *M_U_i_,G_*||*X_S_j__*||*T_G_*′), and checks M_G,U_i__^*^ = ? *M_G,U_i__*. If successful, *U_i_* computes *X_S_j__* = *q_j_* ⊕ *RN_j_* and the session key *K_S_* = *f*((*DID_i_*||*RN_j_*), *X_S_j__*). Then, *U_i_* and *S_j_* successfully realize mutual authentication and have a common session key *K_S_*.

#### 2.1.4. Password Change Phase

This phase provides user *U_i_* to change his/her password by performing the following steps:

Step 1: *U_i_* inserts his smartcard and inputs his/her identity ID_i_^*^, old password pw_i_^*^, and a new password *pw_ni_*.
Step 2: The smart card computes RN_r_^*^ = *h*(pw_i_*) ⊕ *XPW_i_*, HPW_i_^*^ = *h*(pw_i_^*^||RN_r_^*^), X_S_i__^*^ = *C_i_* ⊕ *h*(*ID_s_*||HPW_i_^*^), B_i_^*^ = *h*(HPW_i_^*^ ⊕ X_S_i__^*^), and checks B_i_^*^ = ? *B_i_*. If successful, the smart card computes *HPW_ni_* = *h*(*pw_ni_*||RN_r_^*^), *A_ni_* = *A_i_* ⊕ *h*(HPW_i_^*^||X_S_i__^*^) ⊕ *h*(*HPW_ni_*||X_S_i__^*^), *B_ni_* = *h*(*HPW_ni_* ⊕ X_S_i__^*^), *C_ni_* = X_S_i__^*^ ⊕ *h*(*ID_s_*||*HPW_ni_*), and replaces (*A_i_*, *B_i_*, *C_i_*) with (*A_ni_*, *B_ni_*, *C_ni_*).

### 2.2. Limitations of the Authentication and Key Agreement Scheme of Kim et al.

This subsection addresses the weaknesses of the authentication and key agreement scheme of Kim *et al.* [16], which include: vulnerability to impersonation, lost smartcard and man-in-the-middle attacks; violation of session key security, and failure to protect user privacy.

#### 2.2.1. Security Against Impersonation Attacks

In the scheme of Kim *et al.*, any legitimate user can obtain the sensor node *S_j_*’s secret X_S_j__^*^ after performing the login phase followed by the authentication and key agreement phase. Malicious user 𝒜 can then easily impersonate *S_j_* to communicate with *GWN* and any user *U_i_* by using the following steps:

Step 1: On receiving the message {*DID_i_*, *M_G,S_j__*, *T_G_*} from *GWN*, 𝒜 computes *y_j_′* = *RN_j_′* ⊕ X_S_j__^*^, *z_i_′* = M_G,S_j__^*^ ⊕ *RN_j_′* and *M_S_j_,G_*′ = *h*(z_i_′||X_S_j__^*^||T_j_), where *RN_j_′* is a nonce selected by 𝒜 and *T_j_* is the timestamp. Then 𝒜 sends back {*y_j_′*, *M_S_j_,G_*′, *T_j_*} to *GWN*
Step 2: Next, 𝒜 is authenticated by *GWN* since *GWN* successfully checks $T_{j}$ and *M_S_j_,G_*′ = ? *M_S_j_,G_*, where *RN_j_′* = *y_j_′* ⊕ *X_S_j__*, z_i_′^*^ = M_G,S_j__^*^ ⊕ *RN_j_′*, *M_S_j_,G_*′^*^ = *h*(z_i_′^*^||*X_S_j__*||*T_j_*).
Step 3: Then, 𝒜 computes the session key *K_S_* = *f*((*DID_i_*||*RN_j_′*), *X_S_j__*) shared with *U_i_*. Thus, 𝒜 successfully impersonates *S_j_* to communicate with *GWN* and *U_i_*.

#### 2.2.2. Security against Lost Smart Card Attacks

The malicious user 𝒜 gets (*ID_s_*, *HID_i_*, *h*(·), *A_i_*, *B_i_*, *C_i_*, *XPW_i_*) from *U_i_*’s smartcard. Then 𝒜 can impersonate *U_i_* to communicate with *GWN* and any sensor node *S_j_* by using the following steps:

Step 1: 𝒜 collects previous messages between *U_i_*, *GWN* and *S_j_*^0^, which include (*DID_i_*^0^, *v_i_*^0^, *T_i_*^0^, *HID_i_*, *y_j_*^0^, *y_i_*^0^, *w_i_*^0^, *q_j_*^0^), and has *S_j_*^0^’s secret *X_S_j__*^0^.
Step 2: 𝒜 computes *RN_j_*^0^ = *y_j_*^0^ ⊕ *X_S_j__*^0^, *X_S_i__* = *y_i_*^0^ ⊕ *RN_j_*^0^, *RN_i_*^0^ = *v_i_*^0^ ⊕ *X_S_i__*, *DID_i_′* = *DID_i_*^0^ ⊕ *h*(*X_S_i__||RN_i_*^0^*||T_i_*^0^) ⊕ *h*(*X_S_i__||RN_i_′||T_i_′*), *M_U_i_,G_*′ = *h*(*A_i_||X_S_i__||RN_i_′||T_i_′*) and *v_i_′* = *RN_i_′* ⊕ *X_S_i__*, where *RN_i_′* is a nonce selected by 𝒜 and *T_i_′* is the current timestamp. Then 𝒜 impersonates *U_i_* and sends the authentication request {*DID_i_′*, *M_U_i_,G_*′, *v_i_*′, *T_i_′*, *HID_i_*} to *GWN*.
Step 3: *GWN* successfully authenticates 𝒜 by checking ${T^{\prime }}_{i}$ and M_U_i_,G_^*^ = ? *M_U_i_,G_*′. Next, *GWN* and *S_j_* realize mutual authentication by validating timestamps *T_G_*, *T_j_* and checking M_G,S_j__^*^ = ? *M_G,S_j__*, M_S_j_,G_^*^ = ? *M_S_j_,G_*. Then *GWN* sends back {*y_i_*, *w_i_*, *M_G,U_i__*, *q_j_*, *T_G_′*} to 𝒜, where *M_G,U_i__* = *h*(*DID_i_′*||*M_S_j_,G_*|| *M_U_i_,G_*||*X_S_j__*||*T_G_′*), *w_i_* = z_i_^*^ ⊕ *X_S_i__*, *y_j_* = *RN_j_* ⊕ *X_S_j__*, *q_j_* = *X_S_j__* ⊕ *RN_j_*, and ${T^{\prime }}_{G}$ is the current timestamp.
Step 4: The adversary 𝒜 computes *RN_j_* = *y_j_* ⊕ *X_S_j__* and *X_S_j__* = *q_j_* ⊕ *RN_j_*. Then, 𝒜 successfully has the session key *K_S_* = *f*((*DID_i_′*||*RN_j_*), *X_S_j__*) shared with *S_j_*.

#### 2.2.3. Security against Man-in-the-Middle Attacks

Additionally, a legitimate user 𝒜 has *S_j_*’s secret X_S_j__^*^ and can successfully perform the man-in-the-middle attack by using the following steps:

Step 1: User 𝒜 intercepts the communications between *GWN* and *S_j_*. After receiving the message {*DID_i_*, *M_G,S_j__*, *T_G_*} from *GWN*, 𝒜 forwards it to *S_j_*.
Step 2: On receiving the message {*y_j_*, *M_S_j_,G_*, *T_j_*} from *S_j_*, 𝒜 computes *RN_j_* = *y_j_* ⊕ X_S_j__^*^, *y_j_′* = *RN_j_′* ⊕ X_S_j__^*^, *z_i_′* = M_G,S_j__^*^ ⊕ *RN_j_′* and *M_S_j_,G_*′ = *h*(*z_i_′*||X_S_j__^*^||*T_j_*), and sends {*y_j_′*, *M_S_j_,G_*′, *T_j_*} to *GWN*, where *RN_j_* is a nonce selected by *S_j_* and *RN_j_′* is a nonce selected by 𝒜, respectively
Step 3: *GWN* successfully checks *T_j_*, computes *RN_j_′* = *y_j_′* ⊕ *X_S_j__*, z_i_′^*^ = M_G,S_j__^*^ ⊕ *RN_j_′*, *M_S_j_,G_*′^*^ = *h*(z_i_′^*^||*X_S_j__*||*T_j_*), and checks *M_S_j_,G_*′^*^ = ? *M_S_j_,G_*′. Then, *GWN* computes *M_G,U_i__*′ = *h*(*DID_i_*||*M_S_j_,G_*′|| *M_U_i_,G_*||*X_S_j__*||*T_G_′*), *w_i_′* = z_i_′^*^ ⊕ *X_S_i__*, *y_j_′* = *RN_j_′* ⊕ *X_S_j__*, *q_j_′* = *X_S_j__* ⊕ *RN_j_′* sends {*y_i_′*, *w_i_′*, *M_G,U_i__*′, *q_j_′*, *T_G_′*} to *U_i_*.
Step 4: The smart card successfully checks *T_G_′* and computes *RN_j_′* = *y_j_′* ⊕ *X_S_j__*, z_i_′^*^ = *w_i_′* ⊕ *X_S_i__*, *M_G_*,*S_j_*′ = z_i_′^*^ ⊕ *RN_j_′*, *M_G,U_i__*′^*^ = *h*(*DID_i_*||*M_S_j_,G_*′^*^|| *M_U_i_,G_*||*X_S_j__*||*T_G_′*), and checks *M_G,U_i__*′^*^ = ? *M_G,U_i__*′. Then *U_i_* computes *X_S_j__* = *q_j_′* ⊕ *RN_j_′* and the session key *K_S_′* = *f*((*DID_i_*||*RN_j_′*), *X_S_j__*) shared with 𝒜. *S_j_* computes the session key *K_S_"* = *f*((*DID_i_*||*RN_j_*), *X_S_j__*) shared with 𝒜.

#### 2.2.4. Violation of Session Key Security

Moreover, the legitimate 𝒜 can derive each *RN_j_* by computing *y_j_* ⊕ X_S_j__^*^ and calculate all used session keys *K_S_* = *f*((*DID_i_*||*RN_j_*), *X_S_j__*) of *U_i_* and *S_j_* since 𝒜 has X_S_j__^*^ and *DID_i_*. Then, 𝒜 derives all transmitted secrets between *U_i_* and *S_j_*. Therefore, the scheme of Kim *et al.* violates session key security.

#### 2.2.5. Failure to Privacy Protection of Users

In the scheme of Kim *et al.*, *U_i_*’s identity *ID_i_* is protected with *GWN*’s secret key *K* and hash function *h*(·), and is not revealed. However, the parameter *HID_i_* = *h*(*ID_i_*||*K*) in the request message {*DID_i_*, *M_U_i_,G_*, *v_i_*, *T_i_*, *HID_i_*} from *U_i_* relies on *U_i_*’s *ID_i_* and is constant. An adversary can then easily distinguish whether any two request messages are from the same user using *HID_i_*. Thus, the scheme of Kim *et al.* fails to exhibit data unlinkability, and cannot realize privacy protection of users [17].

## 3. Proposed Authentication and Key Agreement Scheme Using Dynamic Identities for WSNs

This section presents a secure authentication and key agreement scheme based on the scheme of Kim *et al.* [16] for WSNs. The proposed scheme appends a dynamic identity for the user and eliminates constant parameters from the user’s request messages such that any two request messages are independent and indistinguishable. It also encrypts the communicating messages with the temporary secret keys rather than the constant secret keys of users and sensor nodes, and diminishes redundant variables. Additionally, the proposed scheme modifies sensor nodes’ secret keys such that a sensor node cannot derive other sensor nodes’ secret keys. Consequently, an adversary cannot discover the secret keys of users and sensor nodes, and thus used session keys and transmitted secrets. The proposed scheme also has registration, login, authentication & key agreement and password change phases. The password change phase is the same as that of the scheme of Kim *et al.*, and therefore is not presented here.

### 3.1. Registration Phase 

In the registration phase, *U_i_* registers his/her identity and password to *GWN*. Then, *GWN* personalizes a smart card for *U_i_*. Meanwhile, *S_j_* keeps (*SID_j_*, X_S_j__^*^) in its storage before being deployed, where X_S_j__^*^ = *h*(*SID_j_*||*K*):

Step 1: *U_i_* ⇒ *GWN*: {*ID_i_*, *HPW_i_*}
  *U_i_* selects *ID_i_*, password *pw_i_*, a random number *RN_r_*, computes *HPW_i_* = *h*(*pw_i_*||*RN_r_*) and sends {*ID_i_*, *HPW_i_*} to *GWN* via a secure channel.
Step 2: *GWN* ⇒ *U_i_*: *U_i_*′s smartcard
  *GWN* computes *HID_i_* = *h*(*ID_i_*||*K*), *X_S_i__* = *h*(*HID_i_*||*K*), *A_i_* = *h*(*HPW_i_*||*X_S_i__*) ⊕ *HID_i_*, *B_i_* = *h*(*HPW_i_* ⊕ *X_S_i__*), *C_i_* = *X_S_i__* ⊕ *h*(*ID_s_*||*HPW_i_*) and personalizes the smartcard for *U_i_* with the parameters: (*ID_s_*, *h*(·), *A_i_*, *B_i_*, *C_i_*, *TID_i_*). Then, *GWN* sends the smartcard to *U_i_* via a secure channel. *GWN* also stores parameters (*TID_i_*, *TID_i_*°, *HID_i_*) in its storage for *U_i_*, where *TID_i_* is the temporal identity for *U_i_*’s next login and *TID_i_* = *RN_G_*, *RN_G_* is a nonce, and *TID_i_*°= "".
Step 3: *U_i_* computes *XPW_i_* = *h*(*pw_i_*) ⊕ *RN_r_* and inserts *XPW_i_* into his/her smartcard.

### 3.2. Login Phase 

**Figure 2:**
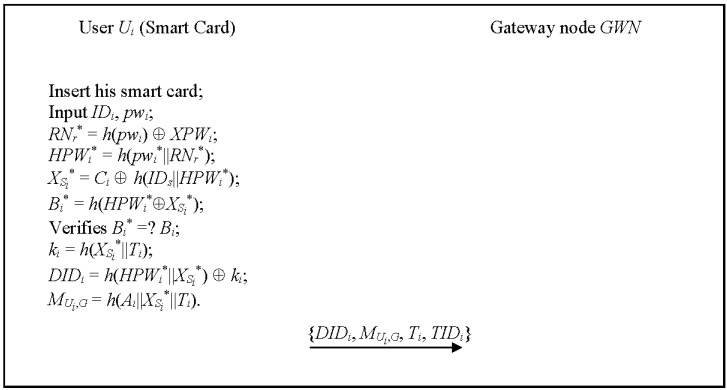
The login phase of the proposed scheme for WSNs.

In this phase, user *U_i_* inserts his/her smart card, inputs his/her identity and password, and sends the service request to *GWN*. Figure 2 illustrates the login phase, which works as follows.

Step 1: $U_{i}$ inserts his/her smart card into a terminal and enters ID_i_^*^ and pw_i_^*^.
Step 2: The smartcard computes RN_r_^*^ = *h*(*pw_i_*) ⊕ *XPW_i_*, HPW_i_^*^ = *h*(pw_i_^*^||RN_r_^*^), X_S_i__^*^ = *C_i_* ⊕ *h*(*ID_s_*||HPW_i_^*^), B_i_^*^ = *h*(HPW_i_^*^ ⊕ X_S_i__^*^) and verifies B_i_^*^ = ? *B_i_*. If unsuccessful, the smartcard aborts this request; otherwise, the smart card computes a temporary secret key *k_i_* = *h*(X_S_i__^*^||*T_i_*), *DID_i_* = *h*(HPW_i_^*^||X_S_i__^*^) ⊕ *k_i_*, *M_U_i_,G_* = *h*(*A_i_*||X_S_i__^*^||*T_i_*), where *T_i_* is the current timestamp. Then the smartcard sends the authentication request {*DID_i_*, *M_U_i_,G_*, *T_i_*, *TID_i_*} to *GWN*.

### 3.3. Authentication and Key Agreement Phase 

**Figure 3:**
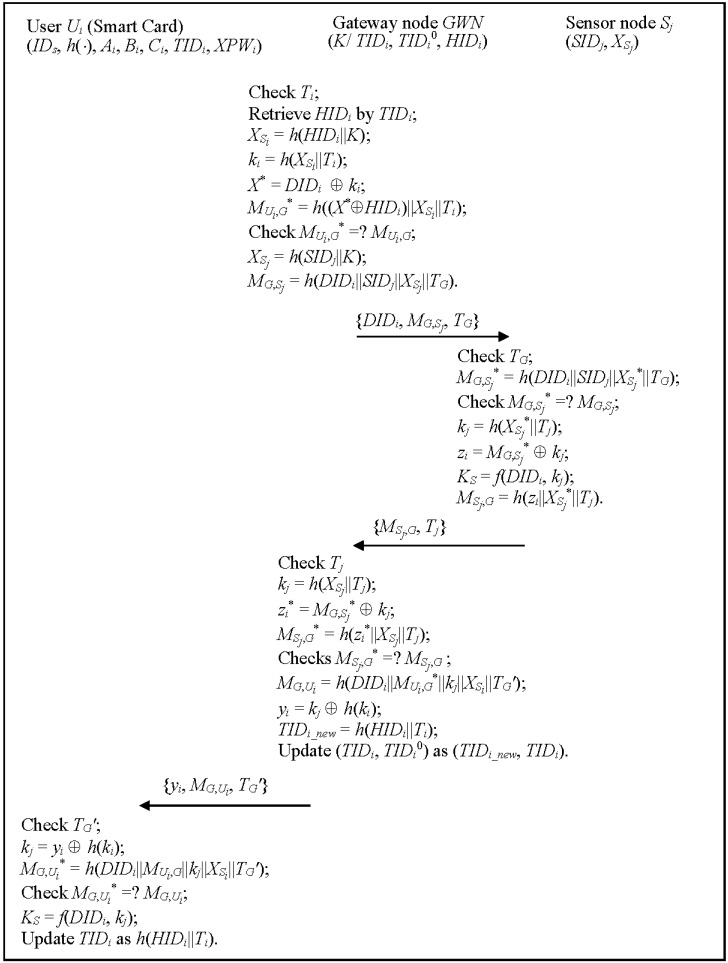
The authentication and key agreement phase of the proposed scheme for WSNs.

This phase enables *U_i_*, *GWN* and *S_j_* to authenticate each other, and to establish a common session key of *U_i_* and *S_j_*. Figure 3 illustrates the authentication and key agreement phase, which works as follows:

Step 1: *GWN* → *S_j_*: {*DID_i_*, *M_G,S_j__*, *T_G_*}
  *GWN* checks the validity of *T_i_*, retrieves *U_i_*,’s information *HID_i_* by using *TID_i_*. If *TID_i_* is not found, then *GWN* retrieves *HID_i_* by using *TID_i_*°. If unsuccessful, *GWN* rejects this service request; otherwise, *GWN* computes *X_S_i__* = *h*(*HID_i_*||*K*), *k_i_* = *h*(*X_S_i__*||*T_i_*), X^*^ = *DID_i_* ⊕ *k_i_*, *M_U_i__*_,G_^*^ = *h*((X^*^ ⊕ *HID_i_*)||*X_S_i__*||*T_i_*) and checks M_U_i_,G_^*^ = ? *M_U_i_,G_*. If successful, *GWN* computes *X_S_j__* = *h*(*SID_j_*||*K*), *M_G,S_j__* = *h*(*DID_i_*||*SID_j_*||*X_S_j__*||*T_G_*) and sends {*DID_i_*, *M_G,S_j__*, *T_G_*} to *S_j_*, where *S_j_* is the nearest sensor node for *U_i_* and *T_G_* is current timestamp.
Step 2: *S_j_* → *GWN*: {*M_S_j_,G_*, *T_j_*}
  *S_j_* checks the validity of *T_G_*, computes M_G,S_j__^*^ = *h*(*DID_i_*||*SID_j_*||X_S_j__^*^||*T_G_*) and checks M_G,S_j__^*^ = ? *M_G,S_j__*. If successful, *S_j_* computes a temporary secret key *k_j_* = *h*(X_S_j__^*^||*T_j_*), *z_i_* = M_G,S_j__^*^ ⊕ *k_j_*, *K_S_* = *f*(*DID_i_*, *k_j_*) and *M_S_j_,G_* = *h*(*z_i_*||X_S_j__^*^||*T_j_*), and sends {*M_S_j_,G_*, *T_j_*} to *GWN*, where *T_j_* is current timestamp.
Step 3: *GWN* → *U_i_*: {*y_i_*, *M_G_*_,_*_U__i_*, *T_G_*′}
  *GWN* checks the validity of *T_j_*, computes *k_j_* = *h*(*X_S_j__*||*T_j_*), z_i_^*^ = M_G,S_j__^*^ ⊕ *k_j_*, M_S_j_,G_^*^ = *h*(z_i_^*^||*X_S_j__*||*T_j_*), and checks M_S_j_,G_^*^ = ? *M_S_j_,G_*. If successful, *GWN* computes *M_G,U_i__* = *h*(*DID_i_*||M_U_i_,G_^*^||*k_j_*||*X_S_i__*||*T_G_*′), *y_i_* = *k_j_* ⊕ *h*(*k_i_*), *TID_i_new_* = *h*(*HID_i_*||*T_i_*), and sends{*y_i_*, *M_G_*_,_*_U__i_*, *T_G_*′} to *U_i_*, where *T_G_*′ is current timestamp. At this time, *GWN* updates (*TID_i_*, *TID_i_*°) as (*TID_i_new_*, *TID_i_*).
Step 4: The smartcard checks the validity of *T_G_*′, and computes *k_j_* = *y_i_* ⊕ *h*(*k_i_*), *M_G_*_,__U__i_^*^ = *h*(*DID_i_*||*M_U_i_,G_*||*k_j_*||*X_S_i__*||*T_G_*′), and checks *M_G_*_,__U_i__^*^ = ? *M_G_*_,_*_U__i_*. If successful, *U_i_* computes the session key *K_S_* = *f*(*DID_i_*, *k_j_*). Then, *U_i_* and *S_j_* successfully realize mutual authentication and have a common session key *K_S_*. Similarly, *U_i_* also updates *TID_i_* as *h*(*HID_i_*||*T_i_*).

## 4. Security Analyses

This section analyzes the security of the proposed authentication and key agreement scheme. The benefits of the proposed scheme provide mutual authentication, session key security, user privacy protection, known-key security and resistance to privileged insider, impersonation and stolen verifier attacks. Since the proposed scheme is based on the scheme of Kim *et al.* [16], the analyses of the resistance to possible attacks, including replay attacks, parallel session attacks, privileged insider attacks and password guessing attacks, closely resemble those for the scheme of Kim *et al.*, and so are not presented here. 

The following descriptions show that the proposed scheme provides the indistinguishability in the Real-or-Random model [17,18,19].

### 4.1. Security Definitions 

#### 4.1.1. AKE Security (Session Key Security)

This definition defines that an adversary cannot effectively distinguish between two messages from a challenger. One message is computed by the real session key and the other one is computed by a random string via an unbiased coin *c*. The adversary selects one message and sends to the challenger. The challenger then decides to return the message computed by the real session key if *c* = 1 or computed by a random string if *c* = 0 by flipping an unbiased coin *c*. The adversary aims to correctly guess the value of the hidden bit *c*. The advantage that an adversary violates the indistinguishability of a scheme is denoted as *Adv^ake^*(𝒜), and is defined as:

*Adv^ake^*(𝒜) = | 2Pr[*E*] − 1 |

where *E* denotes the event that the adversary wins this game. The scheme is AKE-secure if *Adv^ake^*(𝒜) is negligible [17,18,19].

#### 4.1.2. Mutual Authentication (MA) Security

In executing a scheme, the adversary 𝒜 violates mutual authentication if 𝒜 can successfully fake the authenticator *M_U_i_,G_*, *M_G,S_j__*, *M_S_j_,G_* or *M_G,U_i__*. The probability of this event is denoted by *Adv^ma^*(𝒜). The scheme is MA-secure if *Adv^ma^*(𝒜) is negligible [17,18,19].

The Difference Lemma [20] is made used within our sequence of games (SOG), which is described as follows:

**Lemma 1.**  *(Difference Lemma). Let A, B and F be events defined in some probability distribution, and suppose that A∧¬F⇔B∧¬F. Then*

| Pr[*A*] − Pr[*B*] | ≤ Pr[*F*]

### 4.2. Session Key Security

**Theorem 1.**  *The advantage that an adversary breaks the AKE security of the proposed scheme:*

*Adv*^ake^ ≤ 3/2*^l^*^−1^+ 4·*Adv_sk_*

*where* *Adv_sk_ denotes the advantage that an adversary breaks the long-term secret key and l is a security parameter*.

**Proof:**  The proof consists of a sequence of games starting at the game G_0_. Each game G_i_ defines the probability of the event *E_i_* that the adversary wins this game. The first game is the real attack against the protocol and the terminal game G_2_ concludes that the adversary has a negligible advantage to break the AKE security of the proposed scheme. Assume that the challenger 𝒜_1_ attempts to breaks long-term secret keys (*X_S_i__* and *X_S_j__*), and the adversary 𝒜_ake_ is constructed to break the session key security. Then 𝒜_ake_ tries to distinguish the real session key from the random string. The challenger 𝒜_1_ sets up the used parameters, starts simulating the scheme and returns the real session key or a random string to 𝒜_ake_ by flipping an unbiased coin c∈{0,1}. The adversary 𝒜_ake_ outputs its guess bit *c*′ and wins if *c*′ = *c*.

**Game** **G_0_:** This game corresponds to the real attack. By definition, we have:

*Adv^ake^* (𝒜_ake_) *=* |2Pr[*E*_0_]−1| (1)

**Game** **G_1_:** This game transforms game G_0_ into game G_1_ by replacing the long-term secret keys, *X_S_i__* and *X_S_j__*, with two random numbers. Thus, by using Lemma 1, we have:

| Pr[*E*_0_] − Pr[*E*_1_] | ≤ 2·*Adv_sk_* (𝒜_1_) (2)

**Game** **G_2_:** This game transforms game G_1_ into game G_2_ by replacing *k_i_* (= *h*(=*X_S_i__*||*T_i_*)) and *k_j_* (= *h*(*X_S_j__*||*T_j_*)) with two random numbers. Then, games G_1_ and G_2_ are indistinguishable except collisions of a hash function in G_2_. Thus, by using the birthday paradox and Lemma 1, we have:

| Pr[*E*_1_] − Pr[*E*_2_] | ≤ 2×(1/2*^l^*) (3)

**Game** **G_3_:** This game transforms previous game except for replacing *K_S_* with a random number. Similarly, games G_2_ and G_3_ are indistinguishable except collisions of a hash function in G_3_, and thus we have:

| Pr[*E*_2_] − Pr[*E*_3_] | ≤ 1/2*^l^* (4)

Therefore, the probability of the event that 𝒜_1_ outputs 1 when the response message is obtained by using the real session key is equal to the probability of the event that 𝒜_ake_ correctly guesses the hidden bit *c* in game G_2_. Similarly, the probability of the event that 𝒜_1_ outputs 1 when the response message obtained by a random string is equal to the probability of the event that 𝒜_ake_ correctly guesses the hidden bit *c* in game G_3_. All session keys are random and independent, and no information about *c* is revealed. Thus, we have:

Pr[*E*_3_] = 1/2 (5)

Combining Equations (1)–(5), we have:

*Adv^ak^^e^*(𝒜_ake_) ≤ 3/2*^l−^*^1^ + 4·*Adv_sk_* (𝒜_1_)

Then the proof is concluded.

### 4.3. Mutual Authentication

**Theorem 2.**  Let Adv^ma^ be the advantage in violating the mutual authentication of the proposed scheme. Then, Adv^ma^ is negligible, and thus the proposed scheme provides mutual authentication.

**Proof:**  The proof also consists of a sequence of games. The first game G_0_ is the real attack against the proposed protocol and the terminal game G_3_ concludes that the adversary has a negligible advantage to break mutual authentication of the proposed protocol. Assume that *Adv_sk_* denotes the advantage that an adversary breaks the long-term secret keys and *l* is a security parameter. The challenger 𝒜_2_ attempts to break long-term secret keys of the proposed scheme, and the adversary 𝒜_ma_ is constructed to break mutual authentication security for the scheme. The adversary 𝒜_ma_ wins this game if he/she successfully fakes the authenticator *M_U_i_,G_*, *M_G,S_j__*, *M_S_j_,G_* or *M_G,U_i__*.

**Game** **G_0_:** This game corresponds to the real attack. By definition, we have:

*Adv^ma^* (𝒜_ma_)=| 2Pr[*E*_0_] – 1 | (6)

**Game** **G_1_:** This game transforms game G_0_ into game G_1_ by replacing *X_S_i__* and *X_S_j__* with two random numbers. Thus, by using Lemma 1, we have:

| Pr[*E*_0_] − Pr[*E*_1_] | ≤ 2·*Adv_sk_* (𝒜_2_) (7)

**Game** **G_2_:** This game transforms game G_1_ into game G_2_ by replacing *k_i_* and *k_j_* with two random numbers. Thus, by using the birthday paradox and Lemma 1, we have:

| Pr[*E*_1_] − Pr[*E*_2_] | ≤ 2×(1/2*^l^*) (8)

**Game** **G_3_:** This game transforms previous game by replacing the authenticators with random numbers. Similarly, games G_2_ and G_3_ are indistinguishable except collisions of a hash function in G_3_, and thus we have:

| Pr[*E*_2_] − Pr[*E*_3_] | ≤ 4×(1/2*^l^*) (9)

Therefore, the probability of the event that 𝒜_2_ outputs 1 when the authenticator is computed by using the real secret key is equal to the probability of the event that 𝒜_ma_ correctly guesses the hidden bit *c* in game G_2_. Similarly, the probability of the event that 𝒜_2_ outputs 1 when the authenticator obtained by a random string is equal to the probability of the event that 𝒜_ma_ correctly guesses the hidden bit *c* in game G_3_. Since no information on the authenticator is leaked to the adversary, we have:

Pr[*E*_3_] = 1/2 (10)

Combining Equations (6)–(10), we have the advantage that the adversary violates the mutual authentication of the proposed scheme is:

*Adv^ma^*(𝒜_ma_)≤ 4·*Adv_sk_*(𝒜_2_)+ 3/2*^l−^*^2^ (11)

and thus is negligible.

### 4.4. Privacy Protection of Users

**Theorem 3.**  *The proposed scheme provides privacy protection of users*.

**Proof:**  The proposed scheme does not reveal the user’s real identity ID_i_; it replaces the constant temporal identity HID_i_ with a dynamic user identity TID_i_, and eliminates constant parameters from the user’s request messages. Consequently, any two request messages are independent and indistinguishable. The proposed scheme thus exhibits user anonymity, unlinkability and data untrackability [21]. Accordingly, the proposed scheme provides users with privacy protection.

### 4.5. Known-Key Security

**Theorem 4.**  *The proposed scheme provides privacy known-key security*.

**Proof:**  Since the parameters DID_i_ and k_j_ are independent among scheme executions, the session keys K_S_ = f(DID_i_, k_j_) generated in different runs are independent where DID_i_ = h(HPW_i_||X_S_i__) ⊕ h(X_S_i__||T_i_) and k_j_ = h(X_S_j__||T_i_). Accordingly, the proposed scheme provides known-key security.

### 4.6. Resistance to Impersonation Attacks

**Theorem 5.**  *The proposed scheme provides privacy known-key security*.

**Proof:**  An adversary who tries to impersonate U_i_ fails to compute k_i_ = h(X_S_i__||T_i_), DID_i_ = h(HPW_i_||X_S_i__) ⊕ k_i_, M_U_i_,G_ = h(A_i_||X_S_i__||T_i_), and fails to send out the correct request messages {DID_i_, M_U_i_,G_, T_i_, TID_i_} in the login phase without correct ID_i_, pw_i_, X_S_i__ and (ID_s_, h(·), A_i_, B_i_, C_i_, HPW_i_, TID_i_) in U_i_’s smart card, where RN_r_ = h(pw_i_) ⊕ XPW_i_, HPW_i_ = h(pw_i_||RN_r_), and T_i_ is the current timestamp. A failed login is detected by GWN in the authentication and key agreement phase, and thus the proposed scheme withstands impersonation attacks.

### 4.7. Resistance to Stolen Verifier Attacks

**Theorem 6.**  *The proposed scheme withstands stolen verifier attacks*.

**Proof:**  In the proposed scheme, the GWN maintains (TID_i_, TID_i_^0^, HID_i_) in the verifier table for each user U_i_. An adversary who steals a GWN’s verifier table and copies (TID_i_, TID_i_^0^, HID_i_) still fails to compute RN_r_ = h(pw_i_) ⊕ XPW_i_, HPW_i_ = h(pw_i_||RN_r_), X_S_i__ = C_i_ ⊕ h(ID_s_||HPW_i_), k_i_ = h(X_S_i__||T_i_), DID_i_ = h(HPW_i_||X_S_i__) ⊕ k_i_ and M_U_i_,G_ = h(A_i_||X_S_i__||T_i_) without the knowledge of user U_i_’s (ID_i_, pw_i_) and (ID_s_, h(·), A_i_, B_i_, C_i_, XPW_i_, TID_i_) in the smartcard. The adversary fails to send the authentication request {DID_i_, M_U_i_,G_, T_i_, TID_i_} to GWN, and a failure login is detected by GWN. Therefore, the enhanced scheme withstands stolen verifier attacks.

### 4.8. Resistance to Lost Smartcard Attacks

**Theorem 7.**  *The proposed scheme withstands lost smart card attacks*.

**Proof:**  An adversary who steals user *U_i_*’s smartcard and copies the message (*ID_s_*, *h*(·), *A_i_*, *B_i_*, *C_i_*, *XPW_i_*, *TID_i_*) still fails to compute *RN_r_* = *h*(*pw_i_*) ⊕ *XPW_i_*, *HPW_i_* = *h*(*pw_i_*||*RN_r_*), *X_S_i__* = *C_i_* ⊕ *h*(*ID_s_*||*HPW_i_*), *k_i_* = *h*(*X_S_i__*||*T_i_*), *DID_i_* = *h*(*HPW_i_*||*X_S_i__*) ⊕ *k_i_* and *M_U_i_,G_* = *h*(*A_i_*||*X_S_i__*||*T_i_*), and fails to send out the correct authentication request {*DID_i_*, *M_U_i_,G_*, *T_i_*, *TID_i_*} without the correct *ID_i_* and *pw_i_*. Consequently, a failed login is detected by *GWN* in the authentication and key agreement phase, and thus the enhanced scheme withstands lost smartcard attacks.

### 4.9. Resistance to Sensor Node Capture Attacks

**Theorem 8.**  *The proposed scheme withstands sensor node capture attacks*.

**Proof:**  The enhanced scheme eliminates the shared secret key *x_s_* of all sensor nodes and *GWN* in the WSN, and modifies the sensor node *S_j_*’s secret key as *X_S_j__* = *h*(*SID_j_*||*K*). That is, each *S_j_* does not require maintaining *x_s_*. Thus, an attacker 𝒜 who has captured *S_j_′* and obtained (*SID_j_′*, *X_S_j__′*) cannot derive other *S_j_*’s secret key, and also cannot impersonate *U_i_*, *GWN* or other *S_j_*.

## 5. Performance Analyses and Functionality Comparisons

### 5.1. Performance Analyses

Table 2 and Table 3 compare the performance and the simulation time of the proposed scheme with Vaidya *et al.*’s scheme [15], Li *et al.*’s scheme [9] and Kim *et al.*’s scheme [16], where *H* denotes the execution time for a one-way hash function operation, and *X* denotes the execution time for an exclusive-or operation. Table 4 lists our simulation environment, including hardware/software specifications and used algorithms. The proposed scheme involves a user *U_i_*, a sensor node *S_j_*, and a gateway node *GWN*. The user *U_i_* is simulated by using a personal computer, the sensor node *S_j_* is simulated by using a mobile device and the gateway node *GWN* is simulated by using a powerful server, respectively.

**Table 2:** The comparisons of related schemes and the proposed scheme.

		Vaidya *et al.* [15]	Li *et al.* [9]	Kim *et al.* [16]	Our Scheme
	*U_i_*	7H + 7X	9H + 5X	9H + 9X	11H + 5X
Computations	*S_j_*	2H	6H + 4X	3H + 2X	4H + 1X
	*GWN*	6H + 6X	11H + 5X	8H + 8X	10H + 4X
	Total	15H + 13X	26H + 14X	20H + 29X	25H + 10X
Used random numbers		5	4	5	3

**Table 3:** The simulation comparisons of related schemes and the proposed scheme.

Simulation Time (ms)	Vaidya *et al.* [15]	Li *et al.* [9]	Kim *et al.* [16]	Our Scheme
*U_i_*	0.00140	0.00162	0.00180	0.00198
*S_j_*	0.00048	0.00144	0.00072	0.00100
*GWN*	0.00084	0.00143	0.00104	0.00130
Total	0.00272	0.00449	0.00356	0.00428

**Table 4:** Simulation environment.

Hardware/Software Specification		
User *U_i_*	Mainboard	ASUSTeK Computer INC. CM5571
	CPU	Intel Core 2 Quad Q8300 @ 2.50 GHz 2.50 GHz
	Memory	4.00 GB Dual-Channel DDR3 @ 533 MHz
	OS	Windows 7 64-bit SP1
Sensor Node *S_j_*	Mainboard	ASUSTeK Computer INC. UX303LN
	CPU	Intel Core i3/i5/i7 4xxx @ 1.70 GHz
	Memory	4.00 GB Single-Channel DDR3 @ 798 MHz
	OS	Windows 8.1 64-bit
Gateway Node *GWN*	Mainboard	IBM 46W9191
	CPU	Intel Xeon E3 1231 v3 @ 3.40 GHz 3.40 GHz
	Memory	8.00 GB Dual-Channel DDR3 @ 800 MHz
	OS	Windows Server 2008 R2 Standard 64-bit SP1
Used Programming Language and Algorithms		
	C/C++	
	Hash function: SHA-1	

The first comparison item in Table 2 lists the computational cost used in login and authentication-key agreement phases. Vaidya *et al.* [15] requires 15 hash function and 13 exclusive-or operations; Li *et al.* [9] requires 11 hash function and 5 exclusive-or operations; Kim *et al.* [16] requires 11 hash function and 5 exclusive-or operations, and the proposed scheme requires 25 hash function and 10 exclusive-or operations, respectively. The subsequent comparison item is uses random numbers. The proposed scheme requires three random numbers, which is less than that required by related schemes. The comparison item in Table 3 lists the simulation time used in login and authentication-key agreement phases. Although the proposed scheme requires more computations and spends much time in simulation than related schemes, it is still computationally simple and retains low energy consumption. 

### 5.2. Functionality Comparisons

Table 5 compares the functionality of the proposed scheme with that of comparable schemes. The comparison items include resisting possible attacks and providing security requirements. Kim *et al.*’s improved scheme [16] is based on Vaidya *et al.*’s scheme [15], and therefore has the similar security problems. Accordingly, both Vaidya *et al.* [15] and Kim *et al.* schemes [16] fail to withstand possible attacks, including impersonation, lost smartcard and man-in-the-middle attacks. They never provide session key security and protect user privacy. Additionally, Li *et al.*’s scheme [9] fails to withstand impersonation and stolen-verifier attacks, and fail to provide privacy protection. The proposed scheme appends a dynamic identity, eliminates redundant parameters, encrypts the communicating messages with the temporary secret keys, and modifies sensor nodes’ secret keys such that a sensor node cannot derive other sensor nodes’ secret keys, and thus withstands possible attacks and provides privacy protection. Therefore, the proposed scheme provides more functionalities and security properties than other examined schemes, and retains low computational cost.

**Table 5:** The comparisons of the related schemes and the proposed scheme.

	Vaidya *et al.* [15]	Li *et al.* [9]	Kim *et al.* [16]	Our Scheme
Resisting replay attacks	Yes	Yes	Yes	Yes
Resisting impersonation attacks	No	No	No	Yes
Resisting gateway node by passing attacks	No	Yes	Yes	Yes
Resisting parallel session attacks	Yes	Yes	Yes	Yes
Resisting password guessing attacks	Yes	Yes	Yes	Yes
Resisting sensor node capture attacks	No	Yes	Yes	Yes
Resisting man-in-the-middle attacks	No	Yes	No	Yes
Resisting lost smartcard attacks	No	Yes	No	Yes
Resisting privileged-insider attacks	Yes	Yes	Yes	Yes
Resisting stolen-verifier attacks	Yes	No	Yes	Yes
Providing session key security	No	Yes	No	Yes
Providing privacy protection of users	No	No	No	Yes

## 6. Conclusions

This study analyzes the weaknesses of the two-factor authentication and key agreement scheme of Kim *et al.*, which include suffering from impersonation attacks, lost smartcard attacks and man-in-the-middle attacks, violation of session key security, and failure to protect user privacy. An efficient and secure authentication and key agreement scheme for WSNs based on the scheme of Kim *et al.* is proposed. The proposed scheme adopts dynamic identities rather than the constant temporary identity and conceals the user’s constant parameters in login requests, encrypts the communicating messages with temporary secret keys rather than the long-life secret keys of users and sensor nodes, and diminishes redundant variables. Our scheme solves the weaknesses in previous approaches; it provides increased functionality and security properties, making it very suitable for WSNs.
